# Convergence and divergence of molecular mechanisms in Hebbian and homeostatic plasticity

**DOI:** 10.3389/fnsyn.2026.1761008

**Published:** 2026-02-09

**Authors:** Kira M. Feighan, Harshit K. Thakare, Stephen D. Glasgow, Timothy E. Kennedy

**Affiliations:** 1 Department of Neurology and Neurosurgery, Montreal Neurological Institute-Hospital, McGill University, Montréal, QC, Canada; 2 Department of Biological Sciences, Brock University, St. Catharines, ON, Canada

**Keywords:** AMPAR trafficking, Hebbian plasticity, homeostatic scaling, long-term depression, long-term potentiation

## Abstract

The umbrella of synaptic plasticity includes associative, activity-dependent alterations in synaptic strength that are thought to underlie learning and memory, and negative feedback that stabilizes network activity, termed Hebbian and homeostatic plasticity, respectively. These forms of plasticity respond to activity oppositely, and on different spatial and temporal scales. However, despite these fundamental differences, many similar molecular mechanisms are engaged by each form of plasticity to alter synaptic strength. Here, we review molecular mechanisms involved in homeostatic plasticity and compare their involvement in Hebbian plasticity. We focus on synaptic scaling, long-term potentiation, and long-term depression, which are mediated by regulation of post-synaptic amino-3-hydroxyl-5-methyl-4-isoxazole-propionate-type glutamate receptor (AMPARs) accumulation. Addressing synaptic scaffolding, intracellular signaling, cell-adhesion, and secreted factors, we identify mechanisms that appear to be convergent, differentially engaged, and divergent that uniquely regulate homeostatic scaling. These comparisons identify clear gaps to be addressed by future studies that aim to parse the contributions of Hebbian and homeostatic plasticity to regulate AMPAR function.

## 1 Introduction

The phrase “cells that fire together wire together” was coined to reflect long-lasting, activity dependent changes in synaptic strength that are believed to underlie information storage in the brain (Hebb, 1949; Shatz, 1994; Tsien, 2000). The most widely studied experimental example of this is Hebbian synaptic plasticity, which includes both long-term potentiation (LTP) and long-term depression (LTD) (Bliss and Collingridge, 1993; Cooper, 2005; Huganir and Nicoll, 2013). Hebbian forms of plasticity reinforce synaptic activity by either strengthening synapses with correlated pre- and post-synaptic activity (LTP) or weakening synapses with non-correlated activity (LTD) (Bliss and Lomo, 1973; Dudek and Bear, 1992). Among the many molecular changes induced by Hebbian forms of plasticity, both LTP and LTD modulate synaptic strength through up- or down-regulation of post-synaptic AMPAR accumulation, respectively (Huganir and Nicoll, 2013).

Although input-specific Hebbian mechanisms are likely involved at the cellular level to support learning and memory, these forms of plasticity can create a positive feedback loop that if unchecked, would threaten the stability of neural networks (Fernandes and Carvalho, 2016). If Hebbian plasticity is left unregulated, strongly driven synapses would be potentiated without bound while weaker inputs would be progressively silenced or even eliminated (Pozo and Goda, 2010). Consequently, Hebbian plasticity necessitates homeostatic regulation, referred to as homeostatic plasticity, which acts as negative feedback counteracting disruptions to neural network activity and maintaining neuronal firing within a physiological range (Turrigiano and Nelson, 2004; Turrigiano, 2012). In the intact brain, such stabilization is crucial as failures or imbalances in homeostatic plasticity have been linked to neurological disorders including autism spectrum disorder and Alzheimer’s disease (Styr and Slutsky, 2018; Ellingford et al., 2021). While there are numerous forms of homeostatic plasticity, we focus here on synaptic scaling, which refers to a global up- or down-regulation of post-synaptic AMPAR expression to preserve the proportional strength of individual synapses (O’Brien et al., 1998; Turrigiano et al., 1998). This global scaling allows stabilization of neural activity, while maintaining information stored from Hebbian processes that alter the relative weight of individual synapses. Collectively, Hebbian and homeostatic plasticity allow neural networks to remain flexible to adapt to new experiences, yet stable enough to preserve information over time.

The temporal and spatial scales of homeostatic and Hebbian forms of plasticity reveal paradoxical opposite conditions of induction. Whereas Hebbian plasticity is typically induced by brief patterns of activity, homeostatic synaptic scaling is induced by chronic alterations in overall neuronal or network firing activity such as chronic silencing with tetrodotoxin (TTX), which shuts down neuronal activity and induces upscaling, or bicuculline methiodide/picrotoxin (PTX), which increases network activity and induces downscaling (Turrigiano et al., 1998; Matsuzaki et al., 2004). Moreover, in contrast to synapse-specific changes in strength following Hebbian plasticity, changes during synaptic scaling are global and impact the strength of synapses across an entire neuron. At a fundamental level, homeostatic mechanisms act as negative feedback and alter synaptic strength in the opposite direction of neuronal activity, whereas Hebbian mechanisms act to reinforce the state of activity at a synapse (Pozo and Goda, 2010; Turrigiano, 2012; Davis, 2013). Consequently, it is critical to disentangle the contributions of these opposite homeostatic and Hebbian processes and understand how they are integrated within individual neurons and impact neural circuits (Turrigiano, 2017).

**Figure 1:**
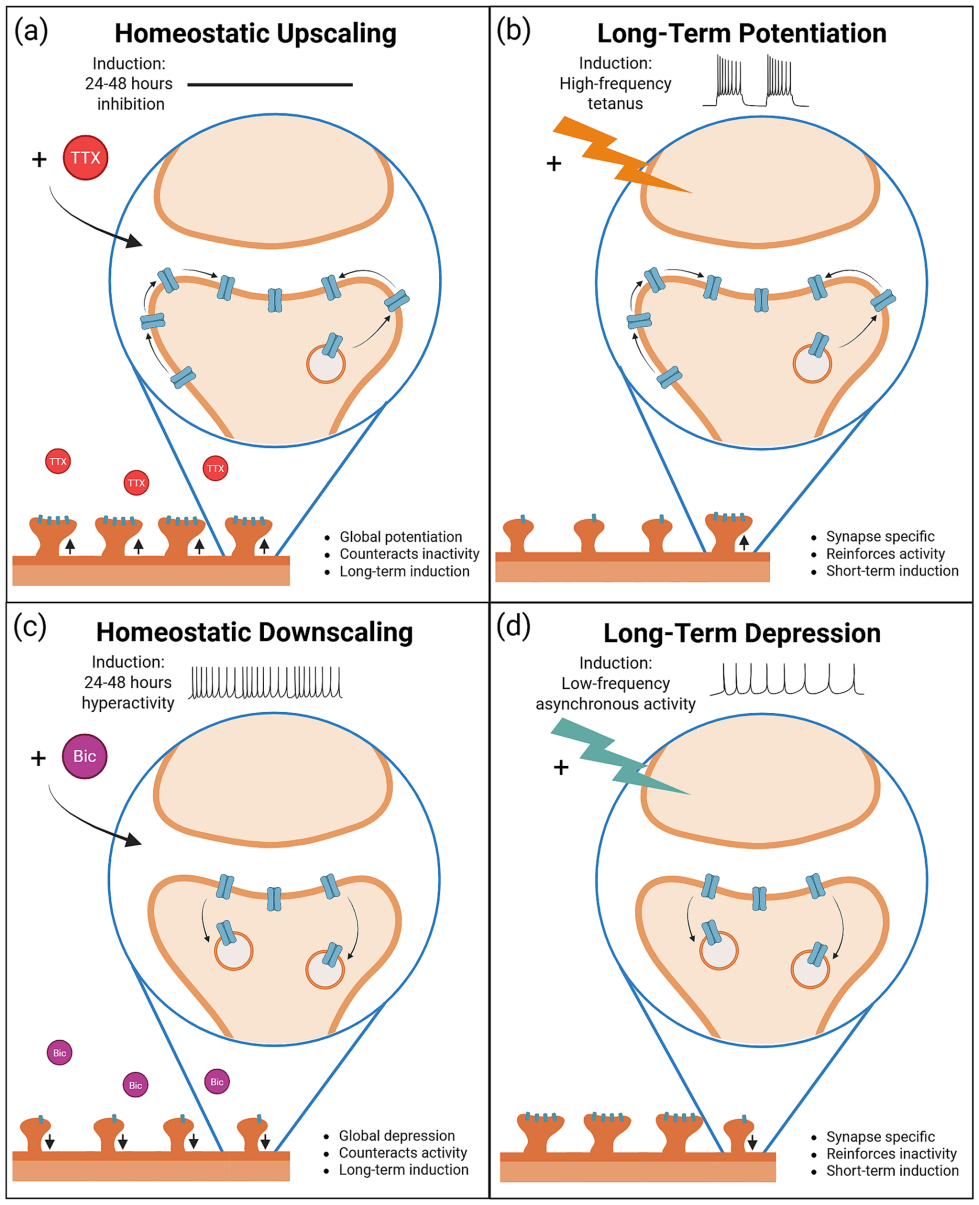
In the broad context of a neuron, homeostatic and Hebbian forms of plasticity appear fundamentally opposite; however, “zooming in” on individual synapses reveals the possibility that mechanisms could be co-opted by both types of plasticity, converging to modulate synaptic strength. **(A,B)** Homeostatic upscaling is induced by chronic inactivity and results in global potentiation at all synapses, whereas LTP is induced by short-term high-frequency tetanus and results in synapse-specific potentiation. Looking at an individual synapse undergoing potentiation during upscaling and LTP shows that both types of plasticity lead to increased synaptic accumulation of AMPARs. **(C,D)** Homeostatic downscaling is induced by chronic hyperactivity and results in global depression at all synapses, whereas LTD is induced by short-term low-frequency stimulation and results in synapse-specific depression. Looking at an individual synapse undergoing depression during downscaling and LTD shows that both types of plasticity lead to decreased synaptic accumulation and endocytosis of AMPARs. Both homeostatic downscaling and LTD may also ultimately result in dendritic spine loss. Created in BioRender. Kennedy, T. (2026) https://BioRender.com/ju86zbs.

Despite substantial differences in induction, both homeostatic scaling and Hebbian LTP/LTD impact post-synaptic strength through regulation of synaptic accumulation of AMPARs at excitatory synapses (O’Brien et al., 1998; Huganir and Nicoll, 2013). An individual synapse undergoing either upscaling or LTP displays increased synaptic AMPAR accumulation, whereas a synapse undergoing either downscaling or LTD displays decreased synaptic AMPAR accumulation (Figures 1A–D). This comparison illuminates the possibility that the distinct induction pathways might eventually converge on the same signaling mechanisms to regulate post-synaptic AMPAR expression, and indeed, a number of studies have identified common mechanisms involved in synaptic modulation during both homeostatic and Hebbian plasticity (Diering et al., 2014; Louros et al., 2014). Critically, a full understanding of convergence and divergence is necessary to understand how homeostatic and Hebbian plasticity co-exist within neural circuits.

Here, we highlight common and divergent mechanisms of homeostatic scaling and Hebbian plasticity with a focus on postsynaptic regulation of AMPARs at excitatory synapses. This review will identify molecules and mechanisms that are similarly engaged in each form of plasticity, as well as those that are clearly distinct, by highlighting convergent pathways, divergent mechanisms, and differentially engaged mechanisms that play distinct roles in homeostatic and Hebbian plasticity. Moreover, we illuminate various unknown mechanisms that lack sufficient evidence to support meaningful comparisons and inform promising directions for future research focused on how homeostatic and Hebbian plasticity can regulate AMPAR accumulation to govern the stability of synaptic connections.

## 2 Potential convergent mechanisms

Despite operating on fundamentally different temporal and spatial scales, both homeostatic scaling and Hebbian plasticity converge on regulation of AMPARs (Bliss and Collingridge, 1993; Diering and Huganir, 2018). These forms of plasticity share parallel mechanisms to manipulate the cell surface distribution of AMPARs. In this section, we discuss common molecules in each form of plasticity, comparing homeostatic upscaling and Hebbian LTP, as well as synaptic depression induced by homeostatic downscaling and LTD. Through this comparison, it becomes apparent that there are numerous shared molecular mechanisms, and that many of these mechanisms share a common characteristic: direct interaction with AMPARs, either through synaptic scaffolding, or via post-translational modification of channel function.

### 2.1 Synaptic scaffolding molecules

#### 2.1.1 Stargazin in upscaling and downscaling

**Figure 2:**
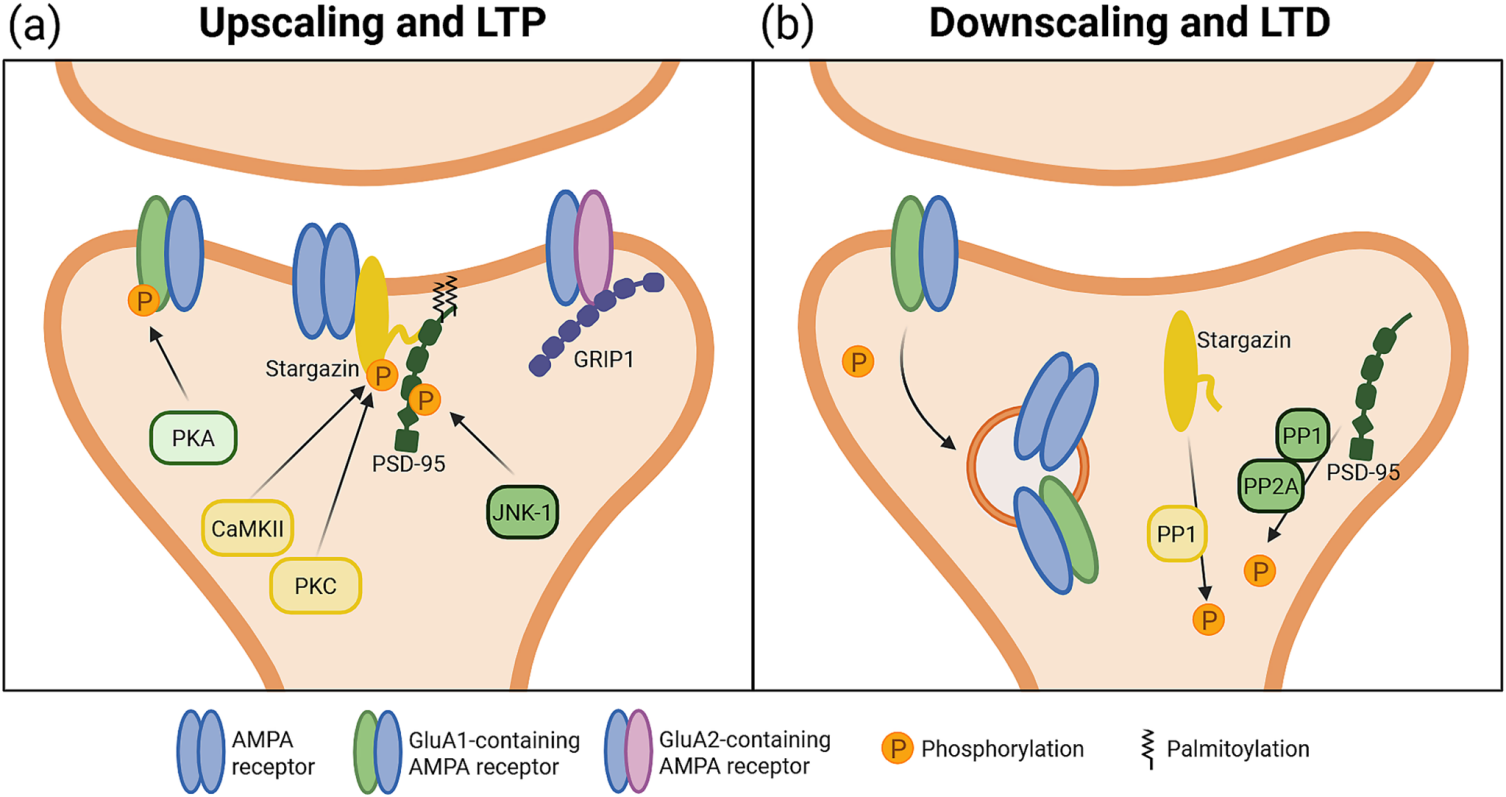
Models illustrating convergent mechanisms of homeostatic and Hebbian plasticity at the post-synapse. **(A)** Synaptic potentiation during both homeostatic upscaling and Hebbian LTP involves CaMKII/PKC-mediated phosphorylation of stargazin, JNK-1-mediated phosphorylation and palmitoylation of PSD-95, GRIP1-mediated trafficking of GluA2-containing AMPARs, and PKA-mediated phosphorylation of GluA1-containing AMPARs. **(B)** Synaptic depression during both homeostatic downscaling and Hebbian LTD involves PP1-mediated de-phosphorylation of stargazin, potentially dissociating stargazin from AMPAR subunit, PP1 and PP2A-mediated de-phosphorylation and de-palmitoylation of PSD-95, and de-phosphorylation of GluA1-containing AMPARs. Although not illustrated, additional AMPAR auxiliary subunits such as cornichons and CKAMPs may also contribute to plastic changes at synapses. Created in BioRender. Kennedy, T. (2026) https://BioRender.com/v6cz31o.

TARPs (transmembrane AMPA receptor-associated proteins) are a family of AMPAR auxiliary subunits that regulate receptor trafficking and function and are critically implicated in multiple forms of synaptic plasticity (Tomita et al., 2003; Payne, 2008; Kamalova and Nakagawa, 2021). Studies of homeostatic scaling have highlighted a role for stargazin, a TARP that directly associates with scaffold protein post-synaptic density-95 (PSD-95) through protein kinase C (PKC)- and calmodulin kinase II (CaMKII)-dependent phosphorylation to stabilize AMPARs at the synapse (Schnell et al., 2002; Opazo et al., 2010). Consistent with a role in homeostatic upscaling, chronic TTX upregulates stargazin expression and phosphorylation, while stargazin knockdown inhibits TTX-mediated increases in surface GluA1-containing AMPARs (Figure 2A) (Louros et al., 2014). Consistent with this, chronic TTX treatment increases PKC activity as well as CaMKIIβ phosphorylation to impact stargazin, and synaptic upscaling is absent in cultures expressing a mutant form of stargazin with the sites targeted by these two kinases rendered phosphodeficient. Together, these findings provide strong evidence that phosphorylation of stargazin is critical for glutamate receptor upscaling.

Stargazin has also been implicated in homeostatic synaptic downscaling. Incubation of dissociated cortical neurons with picrotoxin, a gamma-aminobutyric A (GABA_A_) receptor blocker, results in a broad de-phosphorylation of stargazin within two hours of treatment that is likely mediated by protein phosphatase-1 (PP1), which is activated during prolonged hyperactivity (Figure 2B) (Siddoway et al., 2013; Louros et al., 2018). Moreover, downscaling results in a decrease in dendritic levels of stargazin during the early stages of plasticity. Expression of a constitutively phosphorylated mutant or preventing interaction between stargazin and clathrin adaptors AP-2 and AP-3A completely blocks AMPAR downscaling (Louros et al., 2018). Together, these studies suggest that stargazin phosphorylation acts as a molecular switch during homeostatic scaling, with phosphorylation increasing AMPARs at the plasma membrane and dephosphorylation promoting endocytosis.

Stargazin phosphorylation also acts as a bidirectional switch to gate Hebbian plasticity. N-methyl-D-aspartate glutamate receptor (NMDAR)-mediated LTP induction increases stargazin phosphorylation, and LTP is blocked by phospho-deficient mutations to stargazin at CaMKII and PKC phosphorylation sites or occluded via phospho-mimic mutations, indicating that LTP expression requires CaMKII-mediated phosphorylation of stargazin (Figure 2A) (Tomita et al., 2005). Additional studies show that chemical LTP or high-frequency stimulation-induced activation of CaMKII effectively immobilizes AMPARs at synapses via stargazin phosphorylation by enhancing its ability to bind PSD-95 (Opazo et al., 2010).

In contrast to increased stargazin phosphorylation underlying the expression of NMDAR-mediated LTP, NMDAR-mediated long-term depression (LTD) triggers stargazin dephosphorylation via activation of protein phosphatase 1 (PP1) (Figure 2B) (Tomita et al., 2005). While the specific mechanisms remain unclear, stargazin can interact with clathrin adaptors AP-2 and AP-3A to facilitate AMPAR endocytosis and degradation during NMDA-mediated LTD (Matsuda et al., 2013). Together, these studies suggest that stargazin plays a pivotal role in regulating both homeostatic and Hebbian plasticity based on its phosphorylation state. During upscaling and LTP, PKC and CaMKII activation increase stargazin phosphorylation, to increase synaptic strength, whereas downscaling and LTD cause stargazin dephosphorylation via PP1, contributing to diminished synaptic strength. Further, stargazin interaction with clathrin adaptors AP-2 and AP-3A is required to decrease synaptic strength during both downscaling and LTD.

Stargazin has 9 phosphorylation sites regulated by CaMKII, PKC, and PP1, and consequently it is possible that differential phosphorylation of these sites may mediate different forms of plasticity (Tomita et al., 2005; Louros et al., 2014). However, the striking similarities between homeostatic and Hebbian plasticity suggests that distinct upstream signaling may converge on stargazin to regulate synaptic accumulation of AMPARs through the same mechanism.

#### 2.1.2 PSD-95 in upscaling and downscaling

Stargazin is one of multiple partners that bind to post-synaptic density-95 (PSD-95), a synaptic scaffold that regulates synaptic expression of AMPARs through direct interactions with TARPs (Won et al., 2017). In contrast to stargazin, PSD-95 is necessary, but not sufficient to drive synaptic upscaling. Overexpression of PSD-95 has no effect on miniature post-synaptic current (mEPSC) amplitude, whereas PSD-95 knockdown blocks homeostatic synaptic upscaling of GluA1 AMPARs in mature neuronal cultures (Figure 2A) (Noritake et al., 2009; Sun and Turrigiano, 2011). Different post-translational modifications such as phosphorylation or palmitoylation of PSD-95 can regulate changes in synaptic strength during plasticity. Chronic blockade of activity using TTX triggers increased PSD-95 phosphorylation at serine-295 (S295) that is dependent on Jun N-terminal kinase1 (JNK1), which in turn promotes the accumulation of additional PSD-95 at synaptic sites (Kim et al., 2007; Sun and Turrigiano, 2011). Chronic TTX treatment also induces PSD-95 palmitoylation, which promotes synaptic accumulation of PSD-95 and is essential for synaptic upscaling (Noritake et al., 2009).

Similar to homeostatic synaptic upscaling, PSD-95 is required for expression of homeostatic downscaling, and knockdown of PSD-95 completely blocks GABA_A_R antagonist-mediated decreases in excitatory synaptic activity (Sun and Turrigiano, 2011). However, in contrast to mechanisms underlying upscaling, chronic hyperactivity induces de-phosphorylation of PSD-95 at S295 in a protein phosphatase 1 and 2a (PP1, PP2A) dependent manner and also decreases palmitoylation, which both act to displace PSD-95 from the synapse and promote protein degradation (Figure 2B) (Kim et al., 2007; Chowdhury et al., 2018). This aligns with evidence that, although PSD-95 knockdown blocks downscaling, PSD-95 overexpression also blocks downscaling, suggesting that excess PSD-95 may exert a dominant negative effect (Sun and Turrigiano, 2011).

Comparison to Hebbian forms of plasticity is complicated by studies that test genetic deletion of PSD-95, which can be confounded by developmental effects and altered basal synaptic transmission (Béïque et al., 2006). However, the effect of acute manipulations of PSD-95 on Hebbian plasticity may provide some insight to function. Overexpression of PSD-95 mimics and occludes LTP expression while enhancing LTD, suggesting that increases in PSD-95 expression may saturate basal synaptic transmission and prevent further strengthening (Stein et al., 2003; Ehrlich and Malinow, 2004). Conversely, expression of dominant negative forms of PSD-95 have no effect on basal AMPAR-mediated synaptic transmission, but block the expression of both LTP and LTD, indicating that PSD-95 is critically involved in the expression of both Hebbian and homeostatic synaptic plasticity (Figures 2A,B) (Ehrlich and Malinow, 2004; Xu et al., 2008).

Phosphorylation of PSD-95 at S295 critically regulates Hebbian plasticity, as levels of phosphorylation at this site are increased following induction of LTP and decreased following induction of LTD (Kim et al., 2007). LTP is also correlated with an increase in JNK1 activity, whereas LTD is associated with increased PP1/PP2A activity, suggesting that PSD-95 phosphorylation is regulated by the same signaling cascades during homeostatic and Hebbian plasticity. Moreover, expression of a phospho-mimic mutant results in inhibition of AMPAR internalization following LTD induction, while overexpression of a phospho-deficient S295A mutant has no impact on LTD. This suggests that, like stargazin, the phosphorylation status of PSD-95 at this site may serve as a bidirectional switch: increased phosphorylation at S295 is associated with synaptic potentiation during upscaling and LTP, and de-phosphorylation associated with synaptic depression during downscaling and LTD.

Less is known about the role of PSD-95 palmitoylation in Hebbian plasticity. While levels of PSD-95 palmitoylation are decreased following LTD induction, and overexpression of a palmitoylation-deficient PSD-95 mutant protein prevents LTP, relatively little is known about how fatty acids can impact AMPAR localization and synaptic retention (Ehrlich and Malinow, 2004; Chowdhury et al., 2018). One possibility is that S295 phosphorylation and concurrent palmitoylation of PSD-95 promote its synaptic accumulation and consequently are key processes in the expression of both homeostatic upscaling and LTP. Conversely, dephosphorylation and reduced palmitoylation may destabilize AMPARs at synapses leading to endocytosis and synaptic depression in downscaling and LTD. Consequently, PSD-95 may serve as a convergent mechanism for the expression of any form of plasticity, and modulation of PSD-95 may form part of a critical signaling cascade during plasticity events.

#### 2.1.3 GRIP1 in upscaling

Glutamate receptor interacting protein 1 (GRIP1) is a synaptic PDZ (post-synaptic density-95/Discs large/zona occludens-1) domain-containing protein that directly binds to and regulates trafficking of AMPAR GluA2 subunits to post-synaptic sites (Dong et al., 1999; Osten et al., 2000; Mao et al., 2010; Mejias et al., 2011). Induction of homeostatic upscaling increases the amount of GRIP1 protein as well as its accumulation at synaptic sites to facilitate interactions with GluA2 (Figure 2A) (Gainey et al., 2015; Tan et al., 2015). However, synaptic accumulation of GRIP1 does not require expression of GluA2, suggesting that increased trafficking of GRIP1 is a primary step in the expression of upscaling and does not dependent on AMPAR binding. Overexpression of GRIP1 triggers an upscaling-like synaptic phenotype, leading to an increase in the amplitude of mEPSCs, while shRNA-mediated knockdown prevents upscaling and associated increases in mEPSC amplitude (Gainey et al., 2015). This knockdown is not rescued by a GRIP1 mutant lacking the GluA2 interaction domain, demonstrating a requirement for GRIP1-GluA2 interaction for expression of homeostatic upscaling. The involvement of GRIP1 has also been associated with phosphorylation of GluA2 at Y876, which has been linked to expression of upscaling, as discussed below under “*Divergent mechanisms*.”

Similar to its role in homeostatic upscaling, GRIP1 is recruited to synapses following chemical LTP induction in culture (Figure 2A) (Tan et al., 2020). Further, expression of NMDAR-mediated Hebbian LTP at hippocampal Schaffer collaterals is decreased in mice conditionally lacking GRIP1, suggesting that changes in GRIP1-GluA2 interactions may be important for activity-dependent plasticity. However, in contrast to changes in phosphorylation at Y876 on GluA2 observed in upscaling, NMDAR-dependent LTP is not blocked in phospho-deficient Y876 mice (Yong et al., 2020). While it remains unclear how phosphorylation of Y876 on GluA2 mediates synaptic changes, these findings suggest that GRIP1 contributes to synaptic AMPAR accumulation during both homeostatic and Hebbian plasticity events.

### 2.2 Intracellular signaling

#### 2.2.1 GluA1 phosphorylation in upscaling and downscaling

Recruitment and trafficking of GluA1-containing AMPARs plays a critical role in the expression of plasticity at mature synapses (Diering and Huganir, 2018; Groc and Choquet, 2020). The intracellular carboxy terminus of GluA1 AMPAR subunits is heavily phosphorylated by various kinases including CaMKII, PKA, and PKC. Like other ligand-gated ion channels, phosphorylation of AMPARs can impact single channel conductance as well as trafficking and localization. Consistent with this, phosphorylation of serine 845 (S845) promotes synaptic insertion and reduces endocytosis of AMPARs to maintain GluA1 at the plasma membrane (Man et al., 2007; He et al., 2009; Diering and Huganir, 2018). In addition to promoting the trafficking of GluA1-containing AMPARs to synapses, phosphorylation at S845 on GluA1 by PKA can also increase channel open probability (Banke et al., 2000). Conversely, de-phosphorylation of S845 through calcineurin promotes receptor internalization (Ehlers, 2000; Man et al., 2007; Sanderson et al., 2016). Together, levels of PKA and calcineurin activity regulate surface levels and function of GluA1 containing AMPARs, suggesting that this may serve as a common mechanism to potently regulate the expression of synaptic plasticity.

Phosphorylation of GluA1 at S845 is increased in rat cortical neurons following TTX treatment (Figure 2A) (Diering et al., 2014). Moreover, S845 is required for synaptic upscaling, as both a phospho-deficient knockin mutation (S845A) and knockout of A-kinase anchor protein 5 (AKAP5), the scaffolding protein that links PKA (and calcineurin) with GluA1, is sufficient to block TTX-mediated increases in excitatory synaptic activity (Diering et al., 2014; Sanderson et al., 2018). Enhanced PKA activity or inhibition of calcineurin also promotes synaptic upscaling (Diering et al., 2014). This aligns with findings that lower somatic calcium levels during chronic inactivity reduce calcineurin activation, which promotes upscaling (Kim and Ziff, 2014). Similarly *in vivo*, S845A mutant mice lack experience-dependent homeostatic scaling induced by binocular deprivation (Goel et al., 2011).

Consistent with a role regulating surface distribution of GluA1, S845 phosphorylation is decreased in rat cortical neurons following 24 h treatment to induce downscaling with the GABA_A_ receptor competitive antagonist bicuculline, suggesting that this regulatory site is bidirectionally modified in homeostatic scaling (Figure 2B) (Diering et al., 2014). Reduced phosphorylation at S845 is associated with reduced PKA activity and reduced levels of the AKAP5 catalytic subunit in dendritic spines. However, inhibition of calcineurin surprisingly has no effect on loss of S845 phosphorylation induced by chronic bicuculline, suggesting that decreased S845 phosphorylation during bicuculline-mediated downscaling is due to decreased PKA activity and associated disruption of PKA-AKAP5 coupling, rather than a result of increased calcineurin phosphatase activity (Diering et al., 2014).

GluA1 subunits can also be phosphorylated by PKC and CaMKII at an additional serine site, S831, that has been implicated in increasing single channel conductance during synaptic plasticity (Mammen et al., 1997; Derkach et al., 1999; Kristensen et al., 2011). GluA1 phosphorylated at S831 is enriched in the PSD, suggesting that this site may also contribute to receptor targeting (Diering et al., 2016; Diering and Huganir, 2018). Consistent with a role in synaptic plasticity, phosphomutant mice in which S831 has been replaced with an inactive alanine (S831A) show reduced mEPSC amplitude following visual deprivation, which typically triggers synaptic upscaling (Goel et al., 2011). Moreover, induction of homeostatic downscaling through incubation with bicuculline for 24 h reduces phosphorylation at GluA1 S831 (Diering et al., 2014). However, research using neurons derived from mutant mice with a phospho-deficient S831A mutation found that TTX-induced upscaling was intact, while bicuculline-induced downscaling was diminished. Thus, the role of S831 phosphorylation in homeostatic plasticity remains unclear.

In addition to roles in homeostatic plasticity, GluA1 phosphorylation at both S831 and S845 has been linked to activity-dependent forms of Hebbian plasticity (Barria et al., 1997; Lee et al., 2000). Both phosphorylation sites appear to show compensatory mechanisms in LTP; double S845 and S831 phosphomutants show accelerated decay of LTP expression following theta-burst stimulation, whereas single point mutations of either site alone result in normal LTP expression (Lee et al., 2003, 2010). The balance of kinase/phosphatase activities on PKA/calcineurin targets such as S845 is implicated in LTP as pharmacological or genetic inhibition of calcineurin promotes LTP, and this is attenuated by inhibiting PKA (Figure 2A) (Ikegami et al., 1996; Wang and Kelly, 1996; Winder et al., 1998; Malleret et al., 2001; Zeng et al., 2001).

A number of investigations have begun to examine the interplay of S845 phosphorylation in homeostatic upscaling and Hebbian LTP. Knockdown of the scaffolding protein AKAP5, which limits S845 phosphorylation, blocks homeostatic upscaling and also prevents activity-dependent chemical LTP in cultured rat cortical neurons (Diering et al., 2014). While chemical LTP treatment typically increases surface GluA1 distribution and increases phosphorylation at S845 on GluA1, these effects are occluded by prior incubation with TTX, suggesting that phosphorylation of S845 on GluA1 is a convergent mechanism in both homeostatic upscaling and LTP.

NMDAR-dependent LTD requires S845 dephosphorylation, and S845A mutant mice display a significant deficit in LTD (Figure 2B) (Lee et al., 1998, 2000, 2010; Ehlers, 2000). However, unlike homeostatic downscaling, S845 dephosphorylation during LTD appears to require calcineurin phosphatase activity. Mice expressing mutated AKAP5 without the calcineurin anchoring site show elevated S845 phosphorylation while also showing compromised expression of LTD, suggesting calcineurin anchoring is essential for LTD expression via regulation of S845 (Sanderson et al., 2012). Together, these findings highlight potential shared involvement of S845 dephosphorylation as a convergent mechanism of AMPAR trafficking during synaptic depression that can be triggered by diverse upstream mechanisms such as decreased PKA activity in the case of downscaling, or calcineurin phosphatase activity during LTD.

Unlike S845A mutant mice, mice with an S831A mutation display normal LTD (Lee et al., 2010). However, S831 phosphorylation is reduced following LTD induction at synapses that have been previously potentiated, suggesting that phosphorylation at this site may reflect the history of activation of an individual synapse (Lee et al., 2000). There are many open questions regarding S831 phosphorylation that require being addressed before an appropriate comparison of convergent vs. divergent involvement in homeostatic and Hebbian plasticity can be made.

Post-translational regulation of AMPAR trafficking and function is a complex subject that is only touched on in this review, for more detailed discussion interested readers should see Diering and Huganir (2018).

## 3 Divergent mechanisms

Upstream of the convergent regulation of AMPAR synaptic localization, homeostatic and Hebbian plasticity exist on fundamentally different temporal and spatial scales. These forms of plasticity are induced by opposite neuronal activity environments: homeostatic mechanisms act in opposition to the current activity of the network, whereas Hebbian mechanisms reinforce synaptic activity. These differences necessitate mechanisms that are uniquely engaged by various forms of activity. In this section, we discuss mechanisms that regulate homeostatic scaling but are dispensable for Hebbian plasticity.

### 3.1 Intracellular signaling

#### 3.1.1 GluA2 phosphorylation at Y876 in upscaling and downscaling

Unlike the convergent role of GluA1 phosphorylation, one of the phosphorylation sites on the C-terminus of GluA2, tyrosine 876 (Y876), uniquely regulates homeostatic scaling (Yong et al., 2020). Initial interest in this site came from a study demonstrating reduced amount and activity of the GluA2 tyrosine phosphatase STEP_61_ following prolonged activity blockade (Jang et al., 2015). Investigating this, Yong et al. (2020) found increased phosphorylation at Y876 following TTX-induced upscaling and decreased levels of phosphorylation following bicuculline-induced downscaling (Yong et al., 2020). Moreover, TTX-induced upscaling is prevented in cultured neurons from a phospho-deficient knock-in mouse (GluA2 Y876F). Additionally, Y876 phosphorylation increases the interaction of GluA2 with GRIP1, and Y876F neurons do not exhibit enhanced synaptic accumulation of GRIP1 following TTX treatment. It should be noted that downscaling was not assessed in this study, therefore the contribution of this site to other forms of homeostatic plasticity remains unknown.

However, Yong et al. (2020) did examine activity-dependent Hebbian LTP and LTD, as tyrosine dephosphorylation of GluA2 had been suggested to regulate mGluR-dependent LTD (Moult et al., 2006; Gladding et al., 2009). Surprisingly, GluA2 Y876F mice display normal mGluR-LTD as well as normal NMDAR-LTD and LTP, indicating that GluA2 Y876 phosphorylation is not required for Hebbian plasticity (Yong et al., 2020). Together, this seminal study shows that phosphorylation of GluA2 at Y876 is required for upscaling but is dispensable for Hebbian forms of plasticity. It remains possible that other GluA2 phosphorylation sites are convergently involved, as the GluA2 tyrosine phosphatase STEP_61_ is implicated in both homeostatic and Hebbian plasticity (discussed below under “*Unknown”*).

#### 3.1.2 MSK1 in upscaling

Mitogen- and stress-activated protein kinase 1 (MSK-1) is activated downstream of the ERK1/2 (extracellular-signal-regulated kinase) and p38 MAPK (mitogen activated protein kinase) signaling cascades (Arthur, 2008). MSK1 has multiple substrates, including CREB, which has generated substantial interest in MSK1 as a regulator of synaptic plasticity. ERK1/2 activation of MSK1 is also stimulated downstream of brain derived neurotrophic factor (BDNF) (Arthur et al., 2004; Daumas et al., 2017), which has been implicated in multiple forms of plasticity (discussed below). MSK1 is required for the expression of homeostatic plasticity in hippocampal neurons, as neurons derived from MSK1 kinase-dead mice fail to scale up mEPSC amplitude following chronic TTX treatment, an effect that is rescued by wild-type MSK1 (Corrêa et al., 2012). This was linked to BDNF signaling, as neither application of BDNF, nor inhibition of TrkB receptors influenced mEPSC amplitude in MSK1 kinase dead neurons. Neurons from MSK1 kinase dead mice do not show the expected decrease in Arc/Arg3.1 expression (discussed below) following TTX exposure. The authors suggest that MSK1 activation normally increases Arc/Arg 3.1 transcription, and that under conditions of homeostatic scaling reduced BDNF signaling leads to decreased MSK1 and therefore decreased Arc/Arg3.1 transcription and less AMPAR endocytosis. Validation of this proposed signaling cascade will require further understanding of the role of BDNF in homeostatic plasticity in different cell types (discussed below under “Differentially engaged mechanisms*”*).

This same MSK1 kinase-dead mouse line was used to evaluate the role of MSK1 in Hebbian plasticity (Daumas et al., 2017). This mutant shows reduced CREB phosphorylation after BDNF stimulation and diminished basal synaptic transmission in hippocampal CA1 in response to Schaffer collateral stimulation. However, NMDAR-dependent LTP and mGluR-dependent LTD are intact, suggesting that MSK1 kinase activity is not required for the expression of Hebbian plasticity (Daumas et al., 2017). Together, these studies place MSK-1 as a specific upstream regulator of homeostatic upscaling and link together a signaling pathway that includes BDNF and Arc/Arg 3.1, other regulators that are differentially engaged between upscaling and LTP.

### 3.2 Cell adhesion and transmembrane molecules

#### 3.2.1 β3 integrin in upscaling

Integrins are transmembrane heterodimers composed of *α* and β subunits that connect the extracellular matrix (ECM) to the actin cytoskeleton and mediate cell–cell interactions (van der Flier and Sonnenberg, 2001). The β3 integrin subunit is enriched at synapses and directly binds the cytoplasmic C-terminal of the AMPAR GluA2 subunit (Pozo et al., 2012). Overexpression of β3 integrin is correlated with increased GluA2 abundance and enhanced AMPAR-mediated currents, suggesting that β3 integrin could regulate synaptic plasticity (Cingolani et al., 2008). Indeed, chronic activity blockade increases plasma membrane β3 integrin, and β3 integrin knockout prevents synaptic upscaling in response to chronic TTX treatment in hippocampal neurons and organotypic slices (Cingolani and Goda, 2008; Cingolani et al., 2008).

While many integrins are required for LTP (Chan et al., 2003, 2006; McGeachie et al., 2011), the role of β3 integrin appears to be limited to homeostatic upscaling. Specifically, β3 integrin knockout or ligand disruption in hippocampal slices has no effect on LTP, LTD, or short term synaptic plasticity (McGeachie et al., 2012).

### 3.3 Secreted molecules

#### 3.3.1 TNFα in upscaling

Tumour necrosis factor α (TNFα) is a pro-inflammatory cytokine that is expressed primarily by astrocytes and microglia in the nervous system (Pribiag and Stellwagen, 2014). Early interest in a synaptic role for TNFα came from evidence that exogenous application of TNFα rapidly increases plasma membrane recruitment of GluA2-lacking AMPARs (Beattie et al., 2002; Stellwagen et al., 2005). TNFα is secreted in hippocampal cultures following chronic activity blockade, and *Tnf* knockout cultures and hippocampal slices lack synaptic upscaling (Stellwagen and Malenka, 2006). Chronic activity block promotes TNFα secretion from glia, and wildtype neurons co-cultured with *Tnf* knockout glia fail to show upscaling, whereas *Tnf* knockout neurons co-cultured with wild-type glia present no deficits. In hippocampal slice cultures with conditional TNFα deletion from microglia, homeostatic upscaling is intact, whereas slice cultures with conditional TNFα deletion from astrocytes fail to exhibit upscaling (Heir et al., 2024). These findings indicate that astrocytes are the essential glial source of TNFα for this form of homeostatic plasticity, yet microglia still appear to regulate TNFα modulation of synaptic strength. Low doses of TNFα are only able to increase excitatory synaptic strength in the presence of microglia, while at high concentrations of TNFα, activated pro-inflammatory microglia return excitatory synaptic strength to baseline (Kleidonas et al., 2023). Further, requirements for TNFα in experience-dependent homeostatic plasticity *in vivo* have been demonstrated following monocular deprivation of visual cortex and whisker sensory deprivation of somatosensory cortex (Kaneko et al., 2008; Greenhill et al., 2015). Although the mechanism underlying TNFα regulation of homeostatic scaling is unknown, it has been shown to increase the amount of cell surface β3 integrin, a potential avenue for future investigation (Cingolani et al., 2008).

TNFα is not required to induce changes in surface levels of AMPARs associated with Hebbian plasticity. Hippocampal slices prepared from *Tnf* knockout or TNFα receptor knockout mice display normal LTP and LTD (Stellwagen and Malenka, 2006). While TNFα is not required for LTP, exogenous application of high concentrations of TNFα inhibits LTP, whereas low concentrations promote LTP, suggesting that TNFα may modulate Hebbian plasticity (Tancredi et al., 1992; Cunningham, 1996; Butler et al., 2004; Pickering et al., 2005; Maggio and Vlachos, 2018). Indeed, TNFα has been shown to change the induction threshold for LTP, suggesting that it may mediate metaplasticity (Singh et al., 2019; Heir and Stellwagen, 2020). Metaplastic regulation of LTP by TNFα was demonstrated in studies showing that high-frequency stimulation in hippocampal stratum oriens supresses subsequent LTP expression in the stratum radiatum through TNFα signaling (Singh et al., 2019, 2022). Future work will be required to establish whether a metaplastic effect is related to TNFα regulation of homeostatic plasticity. Yet, the specific recruitment of GluA2-lacking, calcium-permeable AMPARs to the plasma membrane by TNFα provides a plausible mechanistic link between TNFα signaling and metaplastic regulation of activity-dependent plasticity, to shift induction thresholds and increase post-synaptic calcium influx independently of NMDARs.

## 4 Differentially engaged mechanisms

In addition to divergent molecular mechanisms that are uniquely engaged by homeostatic scaling, opposite levels of activity required for the induction of homeostatic and Hebbian plasticity may recruit or regulate the same molecules in different ways. This includes signaling through different receptors or with alternate downstream effects as well as molecules that might be upregulated in one context and downregulated in the other. Here, we review molecular mechanisms that are required for both upscaling and LTP or downscaling and LTD but are differentially regulated or engage different mechanisms of action when comparing these forms of plasticity. The pleiotropic effects of these mechanisms are especially interesting targets for investigating the different temporal and spatial effects of homeostatic and Hebbian plasticity.

### 4.1 Synaptic scaffolding molecules

#### 4.1.1 PICK1 in upscaling

**Figure 3:**
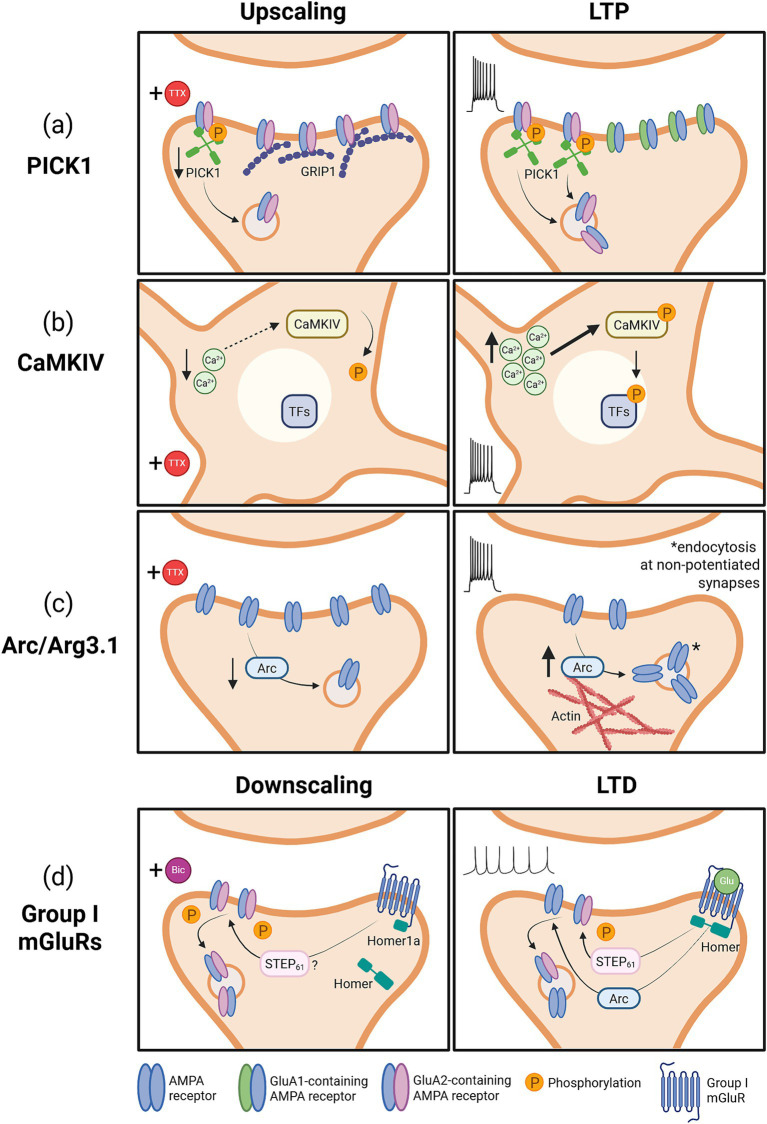
Models illustrating differentially engaged mechanisms underlying homeostatic and Hebbian plasticity. **(A)** PICK1 expression is decreased during homeostatic upscaling, leading to decreased endocytosis of GluA2-containing AMPARs which are stabilized by GRIP1. PICK1 is required for LTP and increases incorporation of GluA2-lacking receptors by promoting endocytosis of GluA2-containing AMPARs. **(B)** Lower intracellular calcium levels during homeostatic upscaling result in decreased activation of CaMKIV. Conversely, increased intracellular calcium during LTP induction activates CaMKIV, triggering it to phosphorylate transcription factors. **(C)** Chronic inactivity during induction of homeostatic upscaling leads to reduced Arc expression, thereby decreasing Arc-mediated endocytosis of AMPARs. Induction of LTP through high-frequency stimulation increases Arc transcription, which is thought to regulate actin polymerization during late-phase LTP, and induce AMPAR endocytosis at non-potentiated synapses to promote hetero-synaptic LTD. **(D)** During homeostatic downscaling, chronic hyperactivity induces Homer1a transcription, which activates group I mGluRs by disrupting their interaction with Homer, leading to downstream reduction of GluA2 phosphorylation (potentially mediated by STEP_61_). During mGluR-mediated LTD, glutamate binding to group I mGluRs activates downstream signaling such as Arc-mediated endocytosis and STEP_61_-mediated GluA2 dephosphorylation. Created in BioRender. Kennedy, T. (2026) https://BioRender.com/hesw5vj.

Protein interacting with C-kinase 1 (PICK1) is a PDZ domain-containing protein that directly competes with GRIP1 (discussed above). PICK1 interacts with activated PKC to phosphorylate GluA2 at the GRIP1 binding site (S880), resulting in dissociation and AMPAR internalization (Matsuda et al., 1999; Chung et al., 2000). In line with enhanced synaptic accumulation of GRIP1, chronic inhibition of activity reduces the amount of PICK1, and TTX-induced upscaling of GluA2 subunit containing AMPARs is occluded in PICK1 knockout or knockdown neuronal cultures, which exhibit increased basal synaptic transmission (Figure 3A) (Anggono et al., 2011).

PICK1 also appears to regulate LTP, although there are discrepancies between studies, and reported effects appear opposite to the influence of PICK1 on homeostatic scaling. PICK1 overexpression was reported to increase AMPAR-mediated synaptic transmission and occlude LTP, while PICK1 knockout prevented LTP in juvenile mice (Terashima et al., 2008), however a subsequent study, using the same mouse line and stimulation parameters, found normal LTP in juvenile PICK1 knockout mice (Volk et al., 2010). It is plausible that increased PICK1 allows greater synaptic incorporation of GluA2-lacking AMPARs in LTP (Figure 3A) (Terashima et al., 2008), however additional studies are required to clarify this mechanism.

It is particularly interesting to note that despite the direct involvement of GRIP1 and PICK1 with each other, GRIP1 seems to similarly contribute to Hebbian and homeostatic plasticity, whereas decreased PICK1 levels promote synaptic upscaling and increased PICK1 levels may promote LTP.

### 4.2 Intracellular signaling

#### 4.2.1 CaMKIV in upscaling

Calcium/calmodulin-dependent protein kinase IV (CaMKIV) is a calcium activated kinase that is primarily localized to the nucleus where it regulates transcription by phosphorylating transcription factors, including CREB (Soderling, 1999). During 24 h TTX-induced chronic activity blockade, somatic calcium levels decrease substantially, leading to reduced activation of CaMKIV (Figure 3B) (Ibata et al., 2008). Inhibition of CaMKIV occludes upscaling, and neuronal transfection with dominant negative or constitutively active CaMKIV is sufficient to mimic upscaling and downscaling, respectively (Joseph and Turrigiano, 2017).

In contrast to the reduced activation following chronic activity deprivation, high-frequency stimulation triggers a transient increase in CaMKIV activation that has been linked to LTP expression (Figure 3B) (Kasahara et al., 2001). Indeed, CaMKIV is required for late-LTP at hippocampal Schaffer collateral synapses, as genetic deletion of CaMKIV or expression of a dominant negative CaMKIV prevents LTP with no effect on LTD (Ho et al., 2000; Kang et al., 2001).

The opposite regulation of CaMKIV activation suggests distinct mechanisms of action in homeostatic verses Hebbian plasticity. It will be important for future research to evaluate the downstream effects of reduced CaMKIV activation that contribute to homeostatic scaling, further distinguishing this from regulation during LTP.

#### 4.2.2 Arc/Arg3.1 in upscaling

Activity related cytoskeleton-associated protein (Arc), otherwise known as activity related gene 3.1 (Arg3.1), is an immediate early gene (IEG) product that rapidly accumulates at synapses following strong synaptic activity (Steward et al., 1998; Steward and Worley, 2001). At synapses, Arc interacts directly with dynamin and endophilin to promote AMPAR endocytosis, thereby decreasing AMPAR-mediated transmission (Chowdhury et al., 2006; Rial Verde et al., 2006). Although Arc mediates AMPAR endocytosis and LTD, it is dispensable for homeostatic downscaling (Shepherd et al., 2006). In upscaling, chronic TTX treatment significantly reduces Arc expression in cultured primary neurons, and Arc overexpression blocks TTX-induced homeostatic upscaling (Figure 3C) (Shepherd et al., 2006). Further, Arc knockout increases basal mEPSC amplitude and occludes upscaling after chronic activity deprivation. *In vivo*, Arc is required for experience-dependent homeostatic plasticity in the visual cortex following dark rearing (Gao et al., 2010).

Paradoxically, Arc transcription is rapidly upregulated following high frequency stimulation-induced LTP, however how this contributes to the expression of LTP remains unclear (Steward et al., 1998). Genetic deletion of Arc in mice has produced conflicting reports, with some knockout studies reporting no effect on LTP (Kyrke-Smith et al., 2021) while others show diminished LTP maintenance (Guzowski et al., 2000; Plath et al., 2006; Messaoudi et al., 2007). Consistent with a role in LTP and in contrast to a role regulating AMPAR endocytosis, Arc regulates actin polymerization in late-LTP (Figure 3C) (Messaoudi et al., 2007). Moreover, Arc has been implicated in heterosynaptic LTD, where it is targeted to non-potentiated synapses to promote AMPAR endocytosis after LTP induction (Figure 3C) (Okuno et al., 2012; Sullivan et al., 2025). This is supported by evidence that induction of Arc translation and the subsequent increase in AMPAR endocytosis is necessary for mGluR-dependent LTD expression (Park et al., 2008; Waung et al., 2008), and increased Arc expression occludes NMDAR-dependent LTD (Rial Verde et al., 2006).

Although Arc is involved in both homeostatic and Hebbian plasticity, distinct mechanisms are likely engaged for down-regulation leading to synaptic potentiation in homeostatic upscaling and up-regulation leading to LTP maintenance. The downstream effects of differential Arc regulation in each context warrants future exploration. Arc is regulated by MSK1 (discussed above), suggesting a possible bifurcating link between its role in various forms of plasticity, however more study is required (Corrêa et al., 2012; Daumas et al., 2017).

#### 4.2.3 Group I mGluRs and Homer 1a in downscaling

Group I mGluRs, comprised of mGluR1 and 5, are metabotropic G-protein coupled glutamate receptors that regulate synaptic strength by modulating the distribution of AMPA and NMDA receptors (Bellone et al., 2008; Lüscher and Huber, 2010). Group I mGluR activity is required for homeostatic downscaling, as demonstrated by impaired downscaling following inactivation of group I mGluRs during chronic bicuculline treatment (Hu et al., 2010). This effect is agonist independent, as bath application of competitive glutamate antagonists failed to prevent downscaling. Group I mGluRs are typically held inactive in the PSD by the synaptic scaffolding protein Homer, and can be activated by intracellular signaling that disrupts the interaction with Homer in addition to typical activation by glutamate (Brakeman et al., 1997; Tu et al., 1998; Ango et al., 2001). Specifically, Homer-mGluR interactions can be displaced by Homer1a, an activity-induced IEG encoding a truncated form of Homer1 that acts as a dominant negative to disrupt effector interactions with full-length Homer isoforms (Clifton et al., 2019). Consistent with this, chronic excitation increases Homer1a transcription, and neuronal overexpression of Homer1a induces downscaling that is blocked by inactivating group I mGluRs (Figure 3D) (Hu et al., 2010). Moreover, Homer1a KO neurons show increased basal cell surface AMPARs and resist downscaling via bicuculline, supporting a model of downscaling in which chronic excitation activates the IEG Homer1a, which in turn liberates group I mGluRs from the Homer scaffold to activate downstream signaling that internalizes AMPARs. While still unknown, it is plausible that reduced GluA2 tyrosine phosphorylation, which is observed in neurons overexpressing Homer1a, mediates this downscaling (Hu et al., 2010).

Group I mGluRs are also critically involved in mGluR-mediated LTD, however the activation of these receptors is distinct from homeostatic downscaling, as Homer1a upregulation is not required for expression of LTD (Bellone et al., 2008; Lüscher and Huber, 2010). Indeed, activation of the group I mGlur, mGluR5, is sufficient to trigger LTD in Homer1a KO mice at Schaffer collateral synapses in the adult hippocampus (Hu et al., 2010). During mGluR-mediated LTD, group I mGluRs are instead activated by synaptic glutamate release (Figure 3D) (Bellone et al., 2008; Lüscher and Huber, 2010). Although the upstream activation of group I mGluRs is different between homeostatic and Hebbian plasticity, this does not rule out convergent downstream signaling pathways.

Two main pathways downstream of mGluR activation during LTD are Arc/Arg3.1 activation and STEP_61_ mediated tyrosine dephosphorylation of AMPARs (Lüscher and Huber, 2010). Arc is rapidly translated downstream of group I mGluR activation and is essential for LTD expression (Park et al., 2008; Lüscher and Huber, 2010). However, as mentioned in the previous section, Arc signaling is dispensable for downscaling, since Arc KO neurons display normal downscaling induced by chronic bicuculline or Homer1a transfection (Shepherd et al., 2006; Hu et al., 2010). Consequently, Arc is an example of divergent signaling downstream of mGluR activation that is required for expression of LTD but not involved in homeostatic downscaling. However, GluA2 dephosphorylation mediated by STEP_61_ is a potential convergent mechanism, and further studies are required to identify the involvement of STEP_61_ in downscaling, as discussed more extensively below under “*Unknown*.”

### 4.3 Cell adhesion and transmembrane molecules

#### 4.3.1 MHC-1 in upscaling

Class 1 major histocompatibility complex (MHC-1) proteins consist of a transmembrane *α* chain and a small extracellular β2-microglobulin and function in the immune system to recognize foreign antigens (Pribiag and Stellwagen, 2014; Thalhammer and Cingolani, 2014). Interest in the neuronal role of MHC-1 s arose from an unbiased screen examining altered RNA expression following long-term activity blockade in the lateral geniculate nucleus (LGN), which detected a decrease in mRNA encoding MHC-1 s (Corriveau et al., 1998). Elaborating on this, cultured hippocampal neurons with reduced MHC-1 fail to upscale in response to chronic inactivity induced by TTX, suggesting involvement in homeostatic plasticity (Goddard et al., 2007).

In contrast, in Hebbian plasticity, MHC-1 deficient mice display enhanced LTP and absence of LTD (Huh et al., 2000). Isolating the mechanisms by which MHC-1 influences synaptic plasticity is difficult, as there are over 70 MHC-1 family members that could be involved (Goddard et al., 2007). Furthermore, it is unclear which immune receptor interacts with MHC-1 in neurons (Shatz, 2009). Despite these unknowns, the evidence that decreased MHC-1 expression blocks upscaling but enhances LTP demonstrates apparent opposite roles in these two plasticity processes. Potential upstream regulators of MHC-1 in the context of plasticity include TNFα, which induces MHC-1 transcription in neurons *in vitro,* and CREB, which increases MHC-1 transcription in the mouse hippocampus when constitutively expressed (Neumann et al., 1997; Barco et al., 2005).

### 4.4 Secreted molecules

#### 4.4.1 BDNF in upscaling

Brain derived neurotrophic factor (BDNF) is an intensively studied neurotrophin that is secreted by neurons in an activity- and calcium-dependent manner (Hartmann et al., 2001; Balkowiec and Katz, 2002). Extracellular BDNF binds tropomyosin receptor kinase B (TrkB) receptors, which initiate numerous downstream signaling cascades (Kowiański et al., 2018). Although BDNF was one of the first molecules identified to regulate homeostatic scaling, its exact role remains poorly understood due to differences between cell types and developmental stages (Turrigiano, 2008). In cultured visual cortex pyramidal neurons, exogenous BDNF application prevents synaptic upscaling following TTX treatment, and BDNF depletion using a TrkB-IgG fusion protein triggers upscaling in cortical cultures, suggesting that BDNF may serve to inhibit the expression of synaptic upscaling (Rutherford et al., 1998). In contrast, exogenous BDNF incubation induces an increase in mEPSC amplitude by increasing surface expression of AMPARs in cultured hippocampal neurons (Bolton et al., 2000; Caldeira et al., 2007). This effect in hippocampal neurons has not been explicitly studied in the context of activity deprivation and homeostatic scaling, however, it seems that the effects of BDNF can be cell-type and context-dependent, triggering an upscaling-like effect in hippocampal neurons while preventing upscaling in cortical neurons. Further complicating its role, BDNF also appears to regulate synaptic scaling of inhibitory interneurons, however discussion of the modulation of these neurons goes beyond the scope of this review (Rutherford et al., 1998; Pozo and Goda, 2010; Fernandes and Carvalho, 2016).

BDNF is secreted at dendritic spines in response to activation of NMDARs and subsequent CaMKII activity (Harward et al., 2016). Early studies showed that BDNF application promotes induction of hippocampal LTP and synaptic accumulation of AMPARs (Figurov et al., 1996; Kovalchuk et al., 2002; Jourdi et al., 2003; Nakata and Nakamura, 2007). Further, treatment of hippocampal slices with BDNF scavenging antibodies attenuates LTP expression, and BDNF null mouse models show LTP impairment at hippocampal Schaffer collateral synapses that is rescued via application of exogenous BDNF (Korte et al., 1995; Figurov et al., 1996; Patterson et al., 1996; Kang et al., 1997; Pozzo-Miller et al., 1999). The exact role of BDNF signaling in LTP is likely multi-tiered and complex, as numerous signaling pathways downstream of BDNF have been implicated in LTP, including PI3K/Akt, MAPK, and PLCγ (Orban et al., 1999; Lin et al., 2001; Minichiello et al., 2002; Thomas and Huganir, 2004).

It is difficult to directly compare the role of BDNF in homeostatic and Hebbian plasticity without more research to address the impact of BDNF on synaptic upscaling. However, given that BDNF blocks upscaling in some cell types while promoting plasticity in others, it appears that BDNF plays a different role in LTP expression, at least in certain cell types.

#### 4.4.2 Retinoic acid, calcineurin, and FMRP in upscaling

Retinoic acid (RA) critically regulates gene expression during neural development (Maden, 2007). Increasing evidence suggests that RA also plays an important role in the adult brain, including regulation of synaptic plasticity. In homeostatic plasticity, strong chronic activity blockade using TTX and the NMDA receptor inhibitor APV (2-amino-5-phosphonovaleric acid) increases RA synthesis, suggesting RA could influence upscaling (Aoto et al., 2008). Indeed, acute RA application induces scaling up in cultured hippocampal neurons and inhibition of RA synthesis prevents upscaling. RA appears to act through the receptor RARα to induce local dendritic protein synthesis and promote synaptic insertion of GluA1-containing AMPARs (Aoto et al., 2008; Maghsoodi et al., 2008; Poon and Chen, 2008; Sarti et al., 2012). Surprisingly, increases in RA synthesis and translation and trafficking of AMPARs to synapses appears to be specific to activity blockade induced by both TTX and APV, and is not observed with TTX alone, suggesting that RA mediates a distinct form of homeostatic scaling (Aoto et al., 2008). It should be noted that this specific form of local dendritic protein synthesis appears to be unique to RA-induced scaling, and is discussed selectively in this section, while the broader role of local protein synthesis in Hebbian plasticity is outside the scope of this review (Pfeiffer and Huber, 2006; Bramham, 2008; Daskin et al., 2025).

Subsequent studies elaborated RA upstream and downstream signaling in homeostatic scaling. Calcineurin, which dephosphorylates GluA1 at S845, as discussed above, also downregulates RA synthesis, and persistent inhibition of calcineurin promotes RA synthesis and synaptic upscaling in neurons (Arendt et al., 2015a). Conversely, upscaling is absent in calcineurin knockout neurons. Calcineurin has no effect on synaptic strength in RAR*α* knockout neurons, implicating calcineurin inhibition upstream of RA in homeostatic scaling. Downstream of RA, fragile X mental retardation protein (FMRP), a dendritically localized mRNA binding protein, regulates dendritic protein synthesis (Soden and Chen, 2010). In *Fmr1* knockout neurons, chronic activity blockade with TTX and APV induces normal RA synthesis but fails to induce local translation of AMPARs, suggesting that FMRP is downstream of RA induced upscaling.

RA has also been implicated in activity-dependent plasticity, though acting through notably distinct receptors and downstream signaling pathways. RA depletion in adult mice or knockout of RAR*β* or RARβ and RARγ impairs LTP (Chiang et al., 1998; Misner et al., 2001). However, conditional deletion of RAR*α* from hippocampal CA1 neurons has no detected impact on Hebbian LTP (Arendt et al., 2015a; Hsu et al., 2019). Comparison of the molecular machinery involved in LTP and RA-mediated synaptic insertion of AMPARs reveals that the two processes use distinct SNARE complex machinery, whereby activity-dependent LTP converges on syntaxin-3 and complexin, while RA regulates syntaxin-4 (Arendt et al., 2015a). This divergence may suggest that fusion of AMPAR-containing vesicles could be directed to different post-synaptic sites: during RA-mediated scaling vesicles may fuse at syntaxin-4-rich membrane sites, and during LTP vesicles may insert at syntaxin-3-rich sites (Arendt et al., 2015a). These studies demonstrate that RA-mediated upscaling and LTP engage distinct AMPAR-trafficking pathways, however other evidence indicates that these two mechanisms still interact. Specifically, acute pre-treatment of hippocampal slices with RA prevents subsequent LTP induction at Schaffer collateral synapses (Arendt et al., 2015b). This effect suggests that both forms of plasticity can saturate post-synaptic strength despite different signaling and trafficking pathways and points to a possible metaplastic interaction between RA-induced upscaling and LTP.

While some studies have linked calcineurin and FMRP to Hebbian plasticity, a calcineurin-RA-FMRP signaling cascade appears to be unique to homeostatic upscaling. Calcineurin inhibition promotes LTP, however this involves calcineurin phosphatase activity at PKA targets and has not been associated with RA signaling (Ikegami et al., 1996; Wang and Kelly, 1996; Winder et al., 1998; Malleret et al., 2001; Zeng et al., 2001). Whether FMRP is involved in LTP is controversial, as several studies have reported normal Schaffer collateral LTP in *Fmr1* knockout mice (Godfraind et al., 1996; Zhang et al., 2009; Bostrom et al., 2015), while others reported impaired LTP (Lauterborn et al., 2007; Hu et al., 2008; Shang et al., 2009; Yun and Trommer, 2011; Tian et al., 2017). It is difficult to comment on the role of FMRP without more conclusive findings, however the studies showing involvement in LTP suggest deficits in phosphoinositide-3-kinase, protein kinase B, and ERK1/2 signaling and do not implicate RA-mediated AMPAR trafficking. Together, these findings highlight discrepancies and emphasize distinct functions for calcineurin, RA, and FMRP signaling during upscaling and the role that each plays in LTP.

## 5 Unknown

The previous sections have detailed molecular mechanisms supported by sufficient research to clearly compare and contrast their roles in homeostatic and Hebbian plasticity. However, a number of detailed mechanisms that contribute to homeostatic plasticity have been investigated that lack comparable studies in the context of Hebbian plasticity. Here, we address each form of plasticity, considering questions that remain to better understand the convergence or divergence of the mechanisms involved.

### 5.1 Synaptic scaffolding molecules

#### 5.1.1 SHANK3 in upscaling and downscaling

SHANK3 is a synaptic scaffolding protein that interacts with a number of PSD proteins, including PSD-95, guanylate kinase associated protein (GKAP), Homer, as well as directly with AMPARs themselves (Uchino and Waga, 2013). Additionally, numerous SHANK3 mutations have been associated with autism spectrum disorder (ASD). Recent evidence demonstrates that SHANK3 influences PSD stability and promotes AMPAR retention which is necessary for plasticity (Jia et al., 2025). SHANK3 knockdown in cultured visual cortical neurons or *in vivo* knockout completely abolishes synaptic upscaling induced by chronic TTX treatment or monocular deprivation, respectively (Tatavarty et al., 2020). Investigating SHANK3 during homeostatic scaling identified two phosphorylation sites (S1615 and S1586) that are consistently de-phosphorylated during upscaling and transiently phosphorylated during downscaling (Wu et al., 2022). In cultured neurons transfected with a phosphomimetic SHANK3 mutant, synaptic upscaling is absent, but downscaling is normal. Conversely, in neuronal cultures transfected with a phosphodeficient mutant, upscaling is normal, but downscaling is absent. These experiments place SHANK3 as a key synaptic scaffold that acts as a bidirectional switch to modulate up and downscaling based on phosphorylation state.

SHANK3 is also required for normal expression of Hebbian LTP, with both SHANK3 heterozygous and homozygous knockout mice displaying altered synaptic transmission and impaired LTP induction and maintenance (Bozdagi et al., 2010; Yang et al., 2012). SHANK3 does not appear to be required for NMDAR-dependent LTD, however expression of *Shank3* ASD mutations in cultured hippocampal neurons prevents mGluR-dependent LTD (Lee et al., 2019). As Shank3 is an essential synaptic scaffold for both homeostatic and Hebbian plasticity it is likely a convergent mechanism, however more evidence is needed for a clear mechanistic understanding as the role of SHANK3 phosphorylation at S1615 and S1586 during LTP and LTD has not been reported. Testing the effects of phosphomimetic and phosphodeficient mutants on LTP and LTD will be crucial to understand the comparative role of SHANK3 in homeostatic and Hebbian plasticity.

### 5.2 Intracellular signaling

#### 5.2.1 CaMKII in upscaling and downscaling

Calcium/calmodulin-dependent protein kinase II (CaMKII) is a large holoenzyme that is the most abundant protein in the post-synaptic density (PSD) (Lisman et al., 2002; Cheng et al., 2006). Once activated by calcium/calmodulin, CaMKII can auto-phosphorylate and support persistent activity after a calcium signal has passed (Miller, 1986). This self-activation has made it an especially intriguing candidate to regulate long-term information storage at synapses (Lisman et al., 2002). Synaptic substrates of CaMKII phosphorylation include GluA1 at S831, leading to increased receptor conductance (Barria et al., 1997; Derkach et al., 1999), and phosphorylation of stargazin, promoting associations between PSD-95 and AMPARs at synapses (Opazo et al., 2010). Indeed, stargazin phosphorylation, as described above, is essential for both homeostatic scaling and Hebbian plasticity.

CaMKII is composed of 6–12 subunits, primarily *α* and *β*, which form homomers or heteromers of variable α/β subunit ratios (Bennett et al., 1983; Brocke et al., 1999). The α subunit responds to higher levels of calcium, while the β subunit is more sensitive and responds to lower levels of calcium (De Koninck and Schulman, 1998; Brocke et al., 1999). This allows the sensitivity of α/β heteromers to be tuned based on subunit composition and indeed the relative expression of α and β subunits is regulated by neural activity (Thiagarajan et al., 2002). Levels of β-CaMKII protein and phosphorylation increase following chronic silencing with TTX, while levels of α-CaMKII increase following chronic activation with bicuculline (Thiagarajan et al., 2002; Louros et al., 2014; Sun et al., 2024), suggesting an involvement in homeostatic plasticity. Indeed, β-CaMKII knockdown prevents homeostatic synaptic accumulation of GluA1 induced by NBQX blockade of AMPARs, and overexpression of β-CaMKII increases GluA1 surface expression (Groth et al., 2011). However, the role of α-CaMKII remains unclear, as the effect of knockdown on bicuculline-induced synaptic downscaling has not been studied.

While CaMKII has many downstream targets, a key target studied in the context of synaptic scaling is GKAP. This family of scaffolding proteins is abundant in the PSD and interacts with PSD-95 and Shanks (Romorini et al., 2004; Shin et al., 2012). When phosphorylated by β-CaMKII, GKAP is recruited to synapses, and consistent with a role in expression of homeostatic plasticity, this association is required for TTX-induced upscaling (Shin et al., 2012). Conversely, GKAP is ubiquitinated and degraded when phosphorylated by α-CaMKII, a process that is required for the expression of bicuculline-induced downscaling. Together, these findings reveal a functional difference between CaMKII isoforms and distinct regulation during scaling.

Early evidence that CaMKII regulates Hebbian LTP came from experiments demonstrating that LTP induction increases CaMKII accumulation and activity in the PSD and overexpression of CaMKII occludes LTP expression (Fukunaga et al., 1993; Lledo et al., 1995; Strack et al., 1997). However, this appears to primarily involve the α subunit, as genetic knockout of α-CaMKII abolishes LTP expression (Silva et al., 1992). β-CaMKII is also required for LTP, however the kinase function of β-CaMKII is not, or can at least be compensated for; mice expressing a mutant form of β-CaMKII lacking the calcium/calmodulin activation site display normal LTP (Borgesius et al., 2011). Instead, β-CaMKII is thought to target α-CaMKII to synapses. Although there is less research on the role of CaMKII in the expression of LTD, selective CaMKII inhibition or knockout of α-CaMKII also blocks NMDAR-dependent LTD (Coultrap et al., 2014). Induction of LTD triggers CaMKII phosphorylation at T305/306, a site not phosphorylated following LTP induction (Cook et al., 2021). Similarly, following LTD induction CaMKII phosphorylates an alternate site on GluA1, S567, which reduces synaptic localization (Coultrap et al., 2014). However, a full understanding of how CaMKII contributes to downscaling and synaptic LTD requires further study.

Together, these studies indicate that CaMKII is critical for both strengthening and weakening synapses in homeostatic and Hebbian contexts. There are clear differences in CaMKII regulation between scaling and Hebbian plasticity, such as specific subunit expression during scaling, which likely reflects upstream differences in calcium dynamics. However, both forms of plasticity require CaMKII to alter synaptic strength, so the question of whether CaMKII can be considered a convergent mechanism lies in whether it has the same downstream activity in each context. The convergent role of stargazin, a CaMKII substrate, points to a level of common downstream CaMKII kinase activity in homeostatic and Hebbian plasticity. However, it must be noted that stargazin phosphorylation can also be mediated by PKC, which is activated following prolonged inactivity that can induce synaptic upscaling (Louros et al., 2014). Further study will be required to unravel the consequences of β-CaMKII and α-CaMKII downstream signaling in scaling, and importantly, to compare CaMKII-mediated phosphorylation of stargazin between upscaling and LTP.

#### 5.2.2 Striatal-enriched protein tyrosine phosphatase 61 (STEP_61_) in upscaling and downscaling

STEP_61_ is a protein tyrosine phosphatase that is enriched at synapses and regulates cell surface distributions of AMPA and NMDA receptors (Braithwaite et al., 2006; Zhang et al., 2008; Kurup et al., 2010). Notably, activation of group I mGluRs triggers STEP_61_ translation, (Zhang et al., 2008), as discussed above in “Differentially engaged mechanisms”, and STEP_61_ activity is regulated by prolonged changes in neuronal activity, suggesting a role in homeostatic scaling. Chronic activity blockade with TTX in cultured rat hippocampal neurons lowers levels of STEP_61_ mRNA and protein, while concomitantly increasing phosphorylation of STEP_61_ at serine-221 (S221), which prevents interaction with all known substrates (Jang et al., 2015). Conversely, chronic hyperexcitation with bicuculline increases STEP_61_ translation, while reducing S221 phosphorylation. These changes in STEP_61_ amount and activity result in increased phosphorylation of GluN2B at Y1472 and GluA2 at Y869, Y873, and Y876 during prolonged silencing, and reduced phosphorylation of the same residues during chronic excitation (Jang et al., 2015). Functionally, TTX-induced scaling is abolished by overexpression of STEP_61_ in cultured hippocampal neurons. This activity-dependent regulation of STEP_61_ aligns with GluA2 phosphorylation at Y876 being necessary for upscaling and reduced during downscaling (discussed above in *“Divergent mechanisms”*) (Yong et al., 2020).

STEP_61_ regulates LTP and mGluR-dependent LTD in a similarly bidirectional manner, however it remains unclear how convergent this may be with STEP_61_ activity in homeostatic plasticity. Induction of LTP triggers rapid, calpain-dependent proteolysis of STEP_61_, and pharmacological inhibition of STEP_61_ enhances LTP magnitude at Schaffer collateral synapses (Saavedra et al., 2019). Moreover, activation of mGluR5 by DHPG to induce LTD increases STEP_61_ translation. This is required for AMPAR endocytosis, which is completely blocked in STEP_61_ KO mice (Zhang et al., 2008). Collectively, these findings suggest that STEP_61_ abundance and activity operates as a molecular switch that mediates changes in synaptic strength during both Hebbian and homeostatic plasticity. Activation of STEP_61_ downstream of group I mGluR activation points to possible convergent regulation between downscaling and LTD. GluA2 phosphorylation at Y876, downstream of STEP_61_, is a unique regulator of upscaling that is dispensable for LTP, however other STEP_61_ targets are likely worth investigating. In summary, significant knowledge gaps remain to be addressed, in particular regarding STEP_61_ downstream targets, to determine whether these effects occur through convergent mechanisms.

#### 5.2.3 MeCP2 in upscaling and downscaling

Methyl CpG-binding protein 2 (MeCP2) is a transcriptional regulator that responds to epigenetic modification by binding methylated DNA to activate or repress transcription (Nan et al., 1997; Yasui et al., 2007; Chahrour et al., 2008; Damen and Heumann, 2013). Mutations in MeCP2 are the most common cause of the X-linked neurodevelopmental disorder Rett syndrome (Wan et al., 1999; Amir and Zoghbi, 2000). MeCP2 was first identified as a regulator of homeostatic downscaling in a screen for transcriptional repressors that are upregulated following chronic bath application of bicuculline in rat hippocampal neuronal cultures (Qiu et al., 2012). Upregulation of MeCP2 was identified to repress GluA2 expression and moreover, knockout or knockdown of MeCP2 blocks synaptic downscaling, indicating a critical role in the expression of downscaling. MeCP2 activity is regulated by phosphorylation at multiple sites, some of which are downstream of neuronal activity dependent calcium influx (Chen et al., 2003; Zhou et al., 2006; Cohen et al., 2011). Cultured hippocampal neurons expressing an MeCP2 phospho-deficient mutation (S421A/424A) fail to reduce excitatory synaptic current amplitude following chronic excitation (Zhong et al., 2012). Downscaling is rescued in these mutants by DHPG application or virally-mediated mGluR5 overexpression, suggesting that upregulation of mGluR5 expression is downstream of MeCP2 phosphorylation during downscaling.

MeCP2 appears to also be required for synaptic upscaling, as acute knockdown or knockout of MeCP2 blocks synaptic upscaling in cultured neurons, and MeCP2 knockout mice lack visual deprivation induced homeostatic changes in the visual cortex (Blackman et al., 2012; Xu and Pozzo-Miller, 2017). The paradoxical requirement of MeCP2 expression for both synaptic upscaling and downscaling is resolved by evidence that differential phosphorylation is required for each form of homeostatic scaling. Specifically, MeCP2 phospho-deficient mutations at S421 and S424 block scaling down but not scaling up, while a phospho-mimic mutation at only S421 has the opposite effect, attenuating scaling up with no effect on scaling down (Zhong et al., 2012; Zhong et al., 2019).

MeCP2-null mice and mouse models of Rett syndrome with MeCP2 mutations also display impaired LTP, NMDAR-LTD, and mGluR-LTD (Asaka et al., 2006; Moretti et al., 2006). Although it is evident that MeCP2 regulates Hebbian plasticity, minimal research has defined the downstream transcriptional changes involved. A key locus of interest is the BDNF promoter which is released from transcriptional repression by phosphorylation of MeCP2 at S421 (Chen et al., 2003; Zhou et al., 2006). Indeed, a phospho-deficient S421 mutant displays enhanced LTP and increased hippocampal *Bdnf* transcription (Li et al., 2011). However, the effect of a phospho-mimic mutant on LTP has not been studied, making it difficult to parse whether MeCP2 impacts upscaling and LTP through the same mechanisms. Moreover, LTD has not been assessed in a MeCP2 phospho-deficient mutant, and how MeCP2 can regulate levels of GluA2 and mGluR5 during LTD remains unclear. This is an active area of research, and future studies to understand the differential effects of MeCP2 on Hebbian and homeostatic plasticity are necessary.

#### 5.2.4 Plk2 in downscaling

The levels of serine/threonine kinase polo-like kinase 2 (Plk2) mRNA and protein are significantly increased following neuronal seizure or high-frequency stimulation (Kauselmann, 1999) as well as by chronic hyperactivity using bicuculline to induce homeostatic downscaling (Pak and Sheng, 2003). Knockdown of Plk2 has been shown to block synaptic downscaling through a number of molecular mechanisms (Seeburg et al., 2008; Evers et al., 2010). Plk2 directly binds *N*-ethylmaleimide–sensitive fusion protein (NSF), a central regulator of SNARE complex disassembly which also directly binds and reduces endocytosis of GluA2 subunits (Evers et al., 2010). Increased Plk2 expression displaces the NSF-GluA2 interaction, resulting in decreased surface GluA2 expression and promoting endocytosis to facilitate downscaling. Moreover, Plk2 serves as a bidirectional regulator of Ras and Rap, small GTPases implicated in memory formation and plasticity (Ye and Carew, 2010). Following chronic inhibitory blockade with PTX, Plk2 phosphorylates numerous guanine nucleotide exchange factors (GEFs) and GTPase-activating proteins (GAPs) leading to an overall downregulation of Ras activity and increase in Rap activity in cultured hippocampal neurons (Lee et al., 2011). These bidirectional changes in Ras/Rap activation result in downstream elimination of mature dendritic spines. Further, Plk2 binds and phosphorylates spine-associated RapGAP (SPAR), which is subsequently ubiquitinated and degraded, resulting in a loss of synaptic PSD-95 clusters and dendritic spine shrinkage (Pak et al., 2001; Pak and Sheng, 2003). SPAR degradation is a critical component of the Plk2 downscaling response, as expression of a degradation-resistant SPAR mutant blocks expression of synaptic downscaling and prevents the loss of mature spines in hippocampal cultures (Seeburg et al., 2008).

Studies investigating a role for Plk2 in Hebbian plasticity are limited, however Ras and Rap bidirectionally regulate LTP and LTD (Zhu et al., 2002, 2005). Ras promotes synaptic delivery of AMPARs during LTP, while Rap mediates AMPAR removal and endocytosis during LTD. Given that Rap contributes to both downscaling and LTD, it is feasible that Rap is a point of convergence for synaptic depression, however this could be clarified by additional studies.

#### 5.2.5 Ubiquitination and AMPAR degradation pathways in downscaling

Two signaling pathways have been described that link the ubiquitination and degradation of GluA1-containing AMPARs to homeostatic downscaling.

Eph proteins are a large family of transmembrane receptor tyrosine kinases that bind membrane tethered ephrins and have well investigated roles in neural development (Himanen and Nikolov, 2003; Kania and Klein, 2016). Chronic bicuculline treatment of cultured cortical neurons activates EphA4, which decreases mEPSC amplitude and reduces synaptic GluA1 content (Fu et al., 2011). EphA4 knockdown blocks bicuculline-induced downscaling, while overexpression of a constitutively active EphA4 scales down mEPSC amplitudes (Peng et al., 2013). EphA4 activation recruits the E3 ubiquitin ligase anaphase promoting complex (APC) and its activator CDH1 (also known as cadherin-1 or E-cadherin) which then poly-ubiquitinates GluA1 and targets it for proteasomal degradation. CDH1 knockdown abolishes GluA1 degradation induced by either EphA4 activation or chronic bicuculline treatment, supporting an essential contribution to downscaling.

An additional pathway implicated in AMPAR degradation involves neural precursor cell-expressed developmentally downregulated gene 4–1 (Nedd4-1), an E3 ubiquitin ligase that ubiquitinates GluA1-containing AMPARs following activation to promote endocytosis and degradation by lysosomes (Schwarz et al., 2010). Chronic treatment of cultured hippocampal neurons with bicuculline significantly increases the amount of Nedd4-1 protein and reduces the amount of the de-ubiquitinating enzyme USP8 (Scudder et al., 2014). Knockdown of Nedd4-1 or overexpression of USP8 blocks bicuculline induced downscaling of mEPSC amplitudes. Together these studies demonstrate essential roles for ubiquitination to direct GluA1 degradation in the expression of homeostatic downscaling.

While these specific ubiquitination pathways have not been studied during LTD, GluA1 ubiquitination contributes to LTD expression. Mice expressing a mutation of the primary ubiquitination site on GluA1 exhibit reduced expression of both NMDAR- and mGluR-dependent LTD (Guntupalli et al., 2023). Expression of NMDAR-dependent LTD also results in lysosomal degradation of the AMPAR GluA1 subunit (Fernández-Monreal et al., 2012). In order to compare with GluA1 regulation during downscaling, it will be important for future studies to examine whether the E3 ubiquitin ligases APC and Nedd4-1 mediate ubiquitination of GluA1 during LTD.

### 5.3 Cell adhesion and transmembrane molecules

#### 5.3.1 Semaphorin 3F (Sema3F) in downscaling

Semaphorins were first characterized as chemorepellent axon guidance cues and are now understood to influence a range of neuronal functions, including synapse formation, pruning, and maturation (Tran et al., 2007; Pasterkamp and Giger, 2009). Class 3 semaphorins are secreted and bind a neuropilin (Nrp) - plexin (Plxn) co-receptor complex to activate downstream signaling. In cultured cortical neurons, chronic hyperactivity increases Sema3F secretion (Wang et al., 2017). Knocking out Sema3F or either of its co-receptors, Nrp2 and PlxnA3, abolishes downscaling induced by chronic bicuculline treatment, demonstrating an essential role for this secreted cue and receptor complex (Wang et al., 2017). Further, Nrp2 associates directly with GluA1, and this interaction is disrupted by Sema3F.

Reports of Sema3F/Npn-2/PlexA3 regulation of Hebbian plasticity are limited to one study that described an miRNA-mediated reduction of Npn-2 protein during LTP (Lee et al., 2012). The interaction of Npn-2 and GluA1 provides particular motivation to further investigate roles for Sema3F signaling in Hebbian LTD and LTP.

#### 5.3.2 N-cadherin and β-catenin in upscaling and downscaling

N-cadherin (CDH2) is a transmembrane protein that mediates Ca^2+^-dependent homophilic adhesions (Takeichi and Abe, 2005; Hirano and Takeichi, 2012). The N-cadherin intracellular domain binds β-catenin, which in turn binds *α*-catenin and links the complex to the actin cytoskeleton. At synapses, the N-cadherin extracellular domain associates with GluA1 and GluA2, allowing N-cadherin/β-catenin to regulate AMPAR trafficking (Nuriya and Huganir, 2006; Saglietti et al., 2007). Conditional knockout of β-catenin from cultured hippocampal neurons results in the loss of both upscaling and downscaling in response to chronic treatment with TTX or bicuculline, respectively (Okuda et al., 2007). β-catenin has several well described intracellular functions, but expression of an N-cadherin mutant lacking the β-catenin binding site similarly diminishes synaptic transmission, supporting a synaptic role for β-catenin downstream of N-cadherin. Expression of a dominant negative N-cadherin prevents scaling up of GluA2 subunits in response to chronic TTX treatment (Vitureira et al., 2011). In contrast, phosphorylation of N-cadherin by Plk2 (discussed above) promotes N-cadherin degradation, which is required for the loss of surface GluA2 during downscaling induced by hyperexcitation (Abdel-Ghani et al., 2023).

N-cadherin also contributes to the late phases of LTP. Following LTP induction, N-cadherin is synthesized and targeted to stimulated spines and newly forming synapses (Bozdagi et al., 2000; Mendez et al., 2010). Blocking N-cadherin adhesion or expression of a dominant negative N-cadherin prevents induction of the late, protein synthesis dependent phase of LTP and the stabilization of potentiated synapses (Bozdagi et al., 2000; Mendez et al., 2010). A specific role for β-catenin in LTP has not been evaluated, however the Wnt/β-catenin signaling pathway is known to regulate LTP, and it has been suggested that this could involve modulation of the interaction between cadherins and F-actin (Chen et al., 2006). There are however discrepancies between studies examining N-cadherin in LTD. One report indicates that N-cadherin ablation has no effect on NMDAR or mGluR-dependent LTD at hippocampal Schaffer-collateral synapses (Bozdagi et al., 2010), while another demonstrates that disruption of the N-cadherin/catenin complex abolishes mGluR-LTD in hippocampal slices (Zhou et al., 2011). It has also been reported that stabilization of N-cadherin surface expression abolishes NMDAR-induced LTD in cultured hippocampal neurons (Tai et al., 2007). Similarly, LTD is abolished in mice expressing a degradation-resistant β-catenin, an effect linked to persistence of cadherins at the plasma membrane (Mills et al., 2014).

Together, these studies identify regulatory roles for N-cadherin/β-catenin in both homeostatic and Hebbian plasticity, but further mechanistic insight is limited. For example, it remains unclear whether direct binding between AMPARs and N-cadherin is critical for homeostatic and Hebbian plasticity. Moreover, it is not known how the dual roles of β-catenin, which is central to both engaging F-actin and Wnt/β-catenin signaling, contributes to changes in synaptic strength.

#### 5.3.3 APP processing in upscaling

The progressive memory loss and synaptic dysfunction in Alzheimer’s disease is characterized by deficits in plasticity that result in neural circuit dysfunction (Styr and Slutsky, 2018). While the etiology of Alzheimer’s disease involves a number of molecules, recent studies have established a role for proteins involved in amyloid precursor protein (APP) processing in homeostatic upscaling and long-term potentiation.

Presenilin-1 (PSEN1) is part of the *γ*-secretase complex which cleaves APP (Bagaria et al., 2022). Mutations in PSEN1 are the most common cause of early onset familial AD. Involvement of PSEN1 in homeostatic plasticity was established using cultured hippocampal neurons derived from PSEN1 knockout mice or mice expressing a familial Alzheimer’s disease linked PSEN1 mutation (*Psen^M146V^*) (Pratt et al., 2011). Neurons from *Psen^M146V^* mice fail to show upscaling of excitatory synaptic current amplitude in response to chronic activity blockade. Moreover, neurons from *Psen^M146V^* mice display elevated ER-mediated calcium activity, increased levels of activated calcineurin, dephosphorylation of GluA1 at S845, and impaired synaptic scaling, suggesting disruption of several mechanisms that could contribute to synaptic dysfunction (Kim et al., 2015).

PSEN1 function has also been implicated in LTP, however the results conflict and the role of PSEN1 and mechanism of action are unclear. Two studies, one examining reduced expression of PSEN1 in heterozygotes, and the other a conditional postnatal PSEN1 knockout, both found diminished LTP (Morton et al., 2002; Dewachter et al., 2008). A third study testing a conditional postnatal PSEN1 knockout, selective for excitatory forebrain neurons, detected normal LTP (Yu et al., 2001). Together, these studies suggest that PSEN1 may be required for LTP, but perhaps not PSEN1 expression in excitatory neurons. In contrast, a number of familial PSEN1 mutations associated with AD enhance LTP expression (Elder et al., 2010). Notably, the *Psen^M146V^* mutation has an age dependent effect with young animals displaying enhanced early LTP and older animals displaying diminished LTP (Auffret et al., 2010). These findings suggest a possible age-related gene-dosage interaction that may differentially impact synaptic plasticity.

BACE1 (β-secretase, beta site APP cleaving enzyme 1) is necessary for the production of the amyloid-β peptide (Cole and Vassar, 2007). Initial evidence that amyloid-β regulates upscaling was derived from finding that BACE1 knockout mice fail to increase excitatory synaptic current amplitude in response to 2 days of dark exposure (Petrus and Lee, 2014). Addition of ectopic amyloid-β to cultured neurons exposed to TTX, or *in vivo* in the visual cortex following visual deprivation, results in aberrant over-scaling (Gilbert et al., 2016). In contrast, dentate granule cells are unable to scale up in response to chronic TTX treatment in organotypic slice cultures derived from APP knockout mice (Galanis et al., 2021). This deficit is replicated by treating wild-type slice cultures with β-secretase or γ-secretase inhibitors and is rescued by addition of amyloid-β. These results provide evidence that amyloid-β production is necessary for expression of homeostatic upscaling.

BACE1, amyloid-β, and APP also impact Hebbian plasticity. Pharmacological inhibition of BACE1 or partial BACE1 depletion in adulthood result in attenuated Schaffer collateral LTP (Filser et al., 2015; Lombardo et al., 2019). This aligns with evidence that amyloid-β is required for normal LTP, as depletion of endogenous levels of amyloid-β attenuates hippocampal LTP (Puzzo et al., 2011). These findings suggest that amyloid-β has a biphasic, dose-dependent effect on LTP, with low concentrations enhancing LTP when perfused over hippocampal slices, and high concentrations impairing hippocampal LTP (Chen et al., 2000; Puzzo et al., 2012). Amyloid-β is not the only proteolytic fragment of APP that regulates LTP. APP knockout mice also display age-related deficits in LTP that are rescued by expression of the *α*-secretase released ectodomain APPsα, and endogenous application of APPsα enhances LTP (Ring et al., 2007; Taylor et al., 2008; Claasen et al., 2009; Moreno et al., 2015).

Together, these studies provide substantial evidence linking APP processing to regulation of both homeostatic and Hebbian plasticity. This identifies both upscaling and LTP as key candidates for understanding synaptic dysfunction in Alzheimer’s disease, however additional studies are required to detail the underlying mechanisms by which APP processing regulates each form of plasticity, and whether this is through convergent or divergent mechanisms.

## 6 Discussion

In this review, we have categorized molecular regulators of homeostatic and Hebbian plasticity, distinguishing between mechanisms that are shared (convergent), unique (divergent), oppositely regulated (differentially engaged), and those lacking comparative evidence (unknown). Through this categorization, it is possible to identify patterns in the interplay between these types of plasticity and areas of research that remain understudied.

Clear patterns emerge when considering convergent molecules, which highlight synaptic scaffolds and post-translational modification of AMPARs to directly regulate their trafficking and functionality. It is of particular interest to note that each molecule that we have described as convergent is located in close physical proximity to AMPARs, but not everything close to AMPARs is convergent.

Molecules classified as divergent are evident when knockout or mutation perturbs one type of plasticity but not the other, however it remains unclear whether this divergence is simply operating upstream of convergent molecules or whether these molecules represent entirely distinct signaling pathways. In principle, divergent mechanisms could function upstream of molecules that participate in convergent regulation of AMPARs. Future studies should aim to explore whether convergent regulation of key synaptic scaffold molecules or GluA1 phosphorylation is required for homeostatic scaling induced by molecules identified here as divergent. However, some mechanisms, such as Y876 phosphorylation of GluA2, appear to constitute a separate but parallel pathway, one that results in the direct covalent modification of an AMPAR subunit, providing an example of a pathway that targets the AMPAR, but is not a convergent target engaged by both homeostatic and Hebbian plasticity.

Unlike divergent mechanisms, molecules classified as “differentially engaged” are required for both homeostatic and Hebbian plasticity but are regulated in opposite directions or play different roles. An obvious explanation for some of these differences is the opposite environmental characteristics of the inducing event, increased or reduced neuronal activity, that triggers each type of plasticity may engage the same molecule but drive it in distinct directions. For example, low somatic calcium levels during silencing reduce CaMKIV activation to induce synaptic upscaling, while high-frequency stimulation to induce LTP increases the concentration of intracellular calcium and activates CaMKIV. This raises the same question of hierarchy: are these differentially engaged factors upstream of convergent mechanisms, or do they regulate distinct pathways?

We conclude with a section addressing unknowns which lack comparative studies between homeostatic and Hebbian plasticity. These provide novel opportunities for productive future research. A comparative, unified approach to study shared and distinct mechanisms will help establish a more comprehensive understanding of the molecular interplay between homeostatic and Hebbian signaling. For example, by systematically testing both forms of plasticity in the same genetic models (e.g., genetic knockouts, knockdowns, or phosphomutants) we can more definitively evaluate convergence versus divergence. A limitation of this review is that it was written primarily through the lens of homeostatic plasticity, and it is important to note that many mechanisms implicated in expression of Hebbian plasticity have yet to be studied in detail in other forms of plasticity. Systematically exploring the mechanisms identified here, as well as those in the wide body of research on LTP and LTD will help resolve the arrangement of parallel and convergent signaling pathways that modulate post-synaptic strength.

## 7 Conclusion

Neurons within neuronal networks employ a range of homeostatic mechanisms to stabilize firing rates in response to developmental or experience-dependent changes in activity. As we have discussed, homeostatic mechanisms counterbalance Hebbian forms of plasticity at single synapses that in turn can help maintain stable network function while preserving synaptic weights that encode information within the network. Ongoing research into the mechanisms that sense neuronal activity, the signaling pathways activated, and how neurons respond is beginning to reveal how homeostatic feedback systems are organized to regulate synaptic function and how these mechanisms interact with synapse-specific Hebbian changes. As homeostatic mechanisms continue to emerge, a key challenge will be to disentangle the molecular machinery involved in tuning synaptic transmission within a neural network to support complex information storage in the nervous system.
